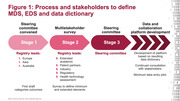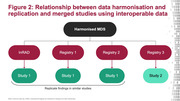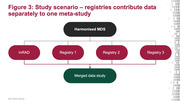# Data harmonisation to foster collaboration between Alzheimer’s disease registries

**DOI:** 10.1002/alz.084775

**Published:** 2025-01-09

**Authors:** Robert Perneczky, Robert Hyde, Johan van Beek, Frank Jessen

**Affiliations:** ^1^ Department of Psychiatry and Psychotherapy, Klinikum der Ludwig‐Maximilians Universität München, Munich Germany; ^2^ German Center for Neurodegenerative Diseases (DZNE, Munich), Feodor‐Lynen‐Strasse 17, 81377 Munich, Germany, Munich Germany; ^3^ Imperial College London, London United Kingdom; ^4^ Sheffield Institute for Translational Neuroscience, University of Sheffield, Sheffield United Kingdom; ^5^ European Alzheimer’s Disease Consortium (EADC), Mannheim Germany; ^6^ TW1 Healthcare Consulting Ltd., London United Kingdom; ^7^ TW1 Healthcare Consulting Ltd, London United Kingdom; ^8^ German Center for Neurodegenerative Diseases (DZNE), Bonn Germany; ^9^ European Alzheimer Disease Consortium., Mannheim Germany; ^10^ Department of Psychiatry, University of Cologne, Medical Faculty, Cologne Germany

## Abstract

Numerous drugs (including disease‐modifying therapies, cognitive enhancers and neuropsychiatric treatments) are being developed for Alzheimer’s and related dementias (ADRD). Emerging neuroimaging modalities, and genetic and other biomarkers potentially enhance diagnostic and prognostic accuracy. These advances need to be assessed in real‐world studies (RWS).

Currently, there are several national and two emerging international ADRD registries that differ in their data requirements. For instance, most existing registries do not routinely capture safety data. Outcome harmonisation would facilitate collaboration between international and national registries and, in turn, support interoperability, and enhance the statistical power and external validity of RWS.

In response, the International Registry for Alzheimer’s Disease and other Dementias (InRAD) convened a Steering Committee of leaders and investigators from registries in Europe, Asia and Australia to define a harmonised minimum dataset (MDS) and extended dataset (EDS) that enables collaboration. A wider stakeholder group, including patient representatives, regulators, payors and industry, will validate the agreed MDS and EDS (Figure 1). The harmonised MDS and EDS will form the basis of data captured in InRAD, which can also form the foundation of collaboration in future RWS within and across registries (Figures 2 and 3).

The harmonised MDS and EDS reflect the needs of two user levels. Firstly, the MDS and EDS should inform differential diagnosis and clinical decision making by presenting longitudinal data in a graphical dashboard summarising important outcomes at the point of care. The harmonised MDS will encompass demographics, functional and cognitive instruments, and rating scales. The harmonised EDS can answer specific questions and/or include additional functional and cognitive instruments to, for example, reflect local clinical practice and patient‐reported outcomes.

Secondly, harmonised MDS and EDS facilitate collaboration between registries to, for example, benchmark, assess efficacy and important safety outcomes, and to inform health technology assessments. The harmonised data sets will be as lean as practical, undergo comprehensive beta‐testing by InRAD and the results shared with stakeholders.

The presentation will explore the background to and need for data harmonisation across registries, the latest iteration of the harmonised MDS and EDS, and InRAD’s overall progress.